# FRL: An Integrative Feature Selection Algorithm Based on the Fisher Score, Recursive Feature Elimination, and Logistic Regression to Identify Potential Genomic Biomarkers

**DOI:** 10.1155/2021/4312850

**Published:** 2021-06-12

**Authors:** Chenyu Ge, Liqun Luo, Jialin Zhang, Xiangbing Meng, Yun Chen

**Affiliations:** ^1^School of Mechanical, Electrical, & Information Engineering, Shandong University, Jinan 250000, China; ^2^Department of Information Management, Peking University, Beijing 100000, China; ^3^Laboratoire de Recherche en Informatique, Paris-Saclay University, Paris 91405, France; ^4^Qufu Institute of Traditional Chinese Medical Health and Rehabilitation, Qufu 273100, China; ^5^The Second Hospital Affiliated to Shandong University of TCM, Jinan 250000, China

## Abstract

Accurate screening on cancer biomarkers contributes to health assessment, drug screening, and targeted therapy for precision medicine. The rapid development of high-throughput sequencing technology has identified abundant genomic biomarkers, but most of them are limited to single-cancer analysis. Based on the combination of Fisher score, Recursive feature elimination, and Logistic regression (FRL), this paper proposes an integrative feature selection algorithm named FRL to explore potential cancer genomic biomarkers on cancer subsets. Fisher score is initially used to calculate the weights of genes to rapidly reduce the dimension. Recursive feature elimination and Logistic regression are then jointly employed to extract the optimal subset. Compared to the current differential expression analysis tool GEO2R based on the Limma algorithm, FRL has greater classification precision than Limma. Compared with five traditional feature selection algorithms, FRL exhibits excellent performance on accuracy (ACC) and F1-score and greatly improves computational efficiency. On high-noise datasets such as esophageal cancer, the ACC of FRL is 30% superior to the average ACC achieved with other traditional algorithms. As biomarkers found in multiple studies are more reliable and reproducible, and reveal stronger association on potential clinical value than single analysis, through literature review and spatial analyses of gene functional enrichment and functional pathways, we conduct cluster analysis on 10 diverse cancers with high mortality and form a potential biomarker module comprising 19 genes. All genes in this module can serve as potential biomarkers to provide more information on the overall oncogenesis mechanism for the detection of diverse early cancers and assist in targeted anticancer therapies for further developments in precision medicine.

## 1. Introduction

Cancers are genomic diseases that cause uncontrolled abnormal cell growth through the constant accumulation of certain genetic mutations [1]. Genes that present specific regulation signals to activate corresponding signaling pathways in cancers are called genomic biomarkers and can be tested by DNA chips [2]. Traditional methods for cancer diagnosis concentrate on abnormalities in human organs and cells, which are intended to be error prone and time consuming as they depend on individual arbitration by an ultrasonic image diagnosis [3, 4]. Precision medicine is defined as the patient-targeted treatment based on the characteristics of genetic abnormalities and biomarkers. Currently, driven by the popularity of precision medicine [5], the goal of targeted therapies for cancers is to track and address biomarkers from multidimensional gene expression data [6].

The DNA chip is one of the applications of microarray technology. Chips can obtain gene expression data by synchronously tracking the expressions of a large number of genes. A gene expression profile has the characteristics of small sample sizes, high dimensionality, and large amounts of noise and redundancy. Feature selection algorithms can identify genomic biomarkers by selecting prominent subsets and classifying the chosen features [7]. The diagnosis and treatment of diverse cancers in biomedicine can be improved and the time required can be reduced by using feature selection algorithms [8].

Feature selection is a classic and effective method to filter out redundant features and form comprehensible models between the eigenvalues and vectors from a given dataset, and it can be categorized into three categories: filter, wrapper, and embedded algorithms [9]. Liang and Vucetic present a filter algorithm for feature selection that uses auxiliary microarray data [7]. Based on divergence or correlation indicators, filter methods function at the intrinsic properties of the dataset to reduce feature redundancy and increase the new classification information [10]. Typically, features with scores above a set threshold are selected. If there is no set of the threshold, the highest-scoring groups are extracted. As open-loop methods, filter methods have good universality and are faster than wrapper and embedded methods [11]. Piao et al. presents a wrapper method of the support vector machine to generate and evaluate subsets of genes [12]. Wrapper methods search feature subsets and perform iterative computation until optimal characteristic features are obtained. In terms of performances on the final model, wrapper methods are better than filter methods, but their calculation costs are high [13]. Embedded feature selection methods integrate the processes of feature selection and model training, which are automatically completed in the same optimization procedure. However, these algorithms are prone to overfitting, as the parameters need to be set to stop the training process at an appropriate time. Sun presents an ensemble scheme for simultaneously reducing dimensionality and extracting features, which greatly improves computational efficiency and accuracy [14]. Normally, an ideal feature selection scheme works as follows: filter algorithms are applied for reducing data dimensionality, and wrapper or embedded methods are further conducted for feature selection [15, 16].

**Figure 1:**
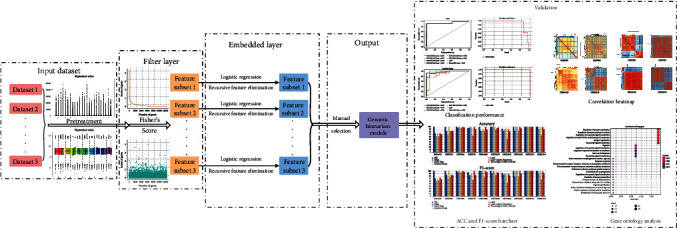
Each rectangle represents a dataset. In the first step, pretreatment on original data is performed. The filter layer performs gene extraction and removes redundancy. The embedded layer is utilized to accurately extract the relevant features from the last step. The selected genes are then grouped to form a genomic biomarker module and are validated through diverse methods.

In biomedical fields, diverse kinds of feature selection algorithms have been applied in extracting specific genomic biomarkers for targeted anticancer therapies [17, 18]. Ensemble classifiers can generally achieve greater precision and generalization ability than individual classifiers [19]. Biomarkers that are more reliable and reproducible, and reveal great potential on clinical application, can be more easily discovered through multiple analyses than through a single study [20]. In order to promote the classification capability of current feature selection methods, this paper creatively proposes a new feature selection algorithm named FRL by combining the advantages of filter methods and embedded methods (Figure 1). This method is implemented as follows: The original data are downloaded, and RMA is utilized to perform based on the R platform. Then, extensive gene extraction is performed on the feature space via a filtering method called Fisher score. Next, Recursive feature elimination and Logistic regression are conjointly utilized to select the relevant features and remove redundant features from the previous dataset in the embedded layer. Furthermore, the selected genes from a total of 10 datasets are grouped together, and ten datasets are compared in pairs and repeated genes on intersections are extracted. Lastly, validation of obtained gene groups is performed, and a genomic biomarker module is constructed through literature review and spatial analyses of functional enrichment and functional pathways.

The full thesis is structured as follows: Section 2 introduces the entire process of our novel FRL algorithm and the relevant validation method. In Section 3, the performance of our FRL is evaluated via four different methods, and esophageal cancer is the representative simulation scenario for comparison with Limma. The selected potential biomarkers in the biomarker module have been validated by diverse analyses in Section 3 as well.  Section 4 and Section 5 present the discussion and conclusion.

## 2. Materials and Methods

### 2.1. Datasets

To validate the classification performance of the FRL and explore more reliable and reproducible potential biomarkers, we apply it to ten microarray gene expression datasets for cancers (GSE1420, GSE3325, GSE7696, GSE9750, GSE16088, GSE18520, GSE18842, GSE54129, GSE56315, and GSE65194). From among all cancer types, we selected the most common types of cancers that have high mortality, namely, lung cancer (21.5%), which is the leading cause of death among men, and breast cancer (15.5%), which is the leading cause for women death [21]. The qualities of these public gene expression datasets regarding diverse cancers are summarized in Table 1. All of the datasets are retrieved from a public repository called the Gene Expression Omnibus (GEO), which can be downloaded from the National Center for Biotechnology Information website (https://www.ncbi.nlm.nih.gov/).

### 2.2. Modulated Characteristics of the Gene Expression Data before Applying FRL

In the pretreatment step illustrated in the first column of Figure 1, we initially downloaded the original files of the gene expression data from the aforementioned database. A background correction method called the robust multiarray average (RMA), which eliminates more noise than other frequently used measures, such as the model-based expression index (MBEI) [35], is applied to the datasets through the bioconductor package on the R platform. The aim of background correction is to remove the effect of the labeled probe on gene expression by binding it to a non-specifically labeled DNA molecule. During this process, because of the diverse regions included in genes, which will directly lead to different probe signals corresponding to certain genes, the number of genes is often slightly less than the number of probes. In allusion to this, we first averaged the probe groups corresponding to a given gene and then choose the largest probe group as the representative to match with the corresponding gene. Then, according to the reference sequences in the public nucleic acid database, a matrix with samples and gene expression data is constructed. Normalization and summarization are performed on matrices for subsequent feature selection. In addition, the gene expression data are transformed into the form of log_2_ to reduce calculation complexity.

### 2.3. Integrative Feature Selection Scheme (FRL) for Identifying Multiple Genomic Biomarkers

Because of the curse of dimensionality, the high-dimensional data from gene expression profiles present challenges for the use of traditional feature selection methods, including overfitting, weak generalization ability, and high variance [36]. The relationship between the samples and features of the cancer datasets is formulated by the following matrix:

(1) $$\begin{array}{c} \left[\begin{array}{c} \left[x_{1}=\left(x_{11,}x_{12,}x_{13,}\cdots x_{1n}\right);y_{1}\right]\\ \left[x_{2}=\left(x_{21,}x_{22,}x_{23,}\cdots x_{2n}\right);y_{2}\right]\\ \left[x_{3}=\left(x_{31,}x_{32,}x_{33,}\cdots x_{3n}\right);y_{3}\right]\\ \vdots \vdots \vdots \\ \left[x_{m}=\left(x_{m1,}x_{m2,}x_{m3,\;}\cdots x_{mn}\right);y_{m}\right] \end{array}\right], \end{array}$$

where *x*_*m*_ is defined as the *m* link of the characteristic vector, and *y*_*m*_ describes the column vector representing the sample categories. Typically, in the machine learning field of a supervised pattern, every *x*_*m*_ is matched with a compatible labeled *y*_*m*_; the feature selection methods in a supervised pattern are aimed at deducing the proper function that can describe the relationship between *x*_*m*_ and *y*_*m*_. Furthermore, the function can suggest the main influencing factor in the original data. Thus, it is vital to propose an effective and robust feature selection method.

To precisely extract genomic biomarkers, we designed an integrative algorithm called FRL. This method is applied on the ten abovementioned cancer datasets. The aim of the FRL algorithm is to focus on dimensionality reduction and sift the optimal subset for further selection of genomic biomarkers. In this method, the scores of each feature are initially computed and ranked based on Fisher score evaluation system; after ranking the scores of genes in descending order, new subsets with high-score genes are formed for each type of cancer. Recursive feature elimination and Logistic regression are cooperatively used to improve the precision of subsets in the next feature selection round. Then, the most ideal subset among the obtained subsets is selected based on relevant measures. Finally, genes are classified into biomarker modules by literature review and spatial analysis of functional enrichment. These processes will be elaborated below in detail.  Procedure 1 presents the entire process of the FRL feature selection method.

#### 2.3.1. Filter Layer: Use Fisher Score to Identify and Delete Redundant Features and Enhance the Quality of Training Datasets

The filter method is implemented as follows: A threshold or correlation coefficient is set as an evaluation criterion by the tester(s), and then the genes of identical quality are extracted based on specific penalty functions and used to form brand-new subsets. This filter mechanism accelerates the speed and performance of classification by converting data with high dimensionality to binary classification problems. In the supervised feature selection field, the formula of Fisher score is defined as follows:

(2) $$\begin{array}{c} F\left(x_{i}\right)=\frac{\sum_{k=1}^{c}n_{k}{\left(\mu_{k}^{j}-\mu^{j}\right)}^{2}}{{\left(\sigma^{j}\right)}^{2}} \end{array}$$

The equation uses *c* to represent the total number of categories. *n*_*k*_ refers to the sample amount of the *j* class.  *μ*_*k*_^*j*^ and *μ*^*j*^ indicate the mean values of the *j* class and the mean values of the current gene expression level, respectively. *σ*^*j*^ denotes the variance value of the *j* class across the whole dataset. Fisher score is employed as the evaluation standard in a filter layer. The scores of each sort can be calculated by equation (2). All genes within the same dataset are scored and arranged in descending order. We identify the inflection point of each cancer category, extract the data before this point to form a subset, and construct matrix *A*_1_. The dimension *D*_1_ of the present matrix is reduced to 1593 averagely from the initial dimension of 21654. This procedure can produce a high-quality output, even with poor computing power.

#### 2.3.2. Embedded Layer: Employ Recursive Feature Elimination and Logistic Regression to Narrow Down the Feature Space

High generalization ability is the core criterion used to identify whether a supervised learning algorithm is effective. Ensemble algorithms can be more effective than a single algorithm to achieve this standard. Thus, here, Recursive feature elimination and Logistic regression are jointly utilized to traverse the whole feature space. In this step, the iteration pace *P* varies from 2 to 5 and is established based on the dimensionality *D*_1_ of each cancer. We set 5 for a dataset that has a dimension of over 2000, 3 for a dataset that has a dimension of over 1000 but less than 2000, and 2 for a dataset that has a dimension of less than 1000. Simultaneously, we divide matrix *A*_1_ into *K* pieces to perform cross validation. The index *K* is set based on the dimensionality *D*_1_ of either data set. If *K* is set as 4, the whole dataset will be divided into 4 pieces. The *K*-fold cross validation will orderly extract one as a test set among 4 sets, leaving the other 3 pieces as a training set for the training model and classifier.

Recursive feature elimination is a typical backward reduction algorithm. However, it cannot be used alone and must be implemented with appropriate classifiers. Recursive feature elimination processes as follows: Firstly, certain classifiers are combined with Recursive feature elimination to train the *A*_1_′_train_. Next, the value of each feature is calculated and irrelevant features are eliminated. Then, the dataset is automatically reconstructed and values for features are calculated again until the optimal feature subsets are obtained.

Logistic regression is applied as the classifier for Recursive feature elimination to determine the probability in each category. Logistic regression is a generalized linear algorithm on binary classification. It is based on the linear regression model and sigmoid function. Hypothesize the linear function as follows:

(3) $$\begin{array}{c} g\left(x\right)=\omega^{T}x+b. \end{array}$$

The sigmoid function can be defined by formula (4), and the logistic regression can be defined by formula (5):

(4) $$\begin{array}{c} \varphi \left(j\right)=\frac{1}{1+e^{-j}}, \end{array}$$

(5) $$\begin{array}{c} \varphi \left(x\right)=\frac{1}{1+e^{-\omega^{T}\;x+b}}. \end{array}$$

The cost function, which can be used to measure the quality of the Logistic regression model is listed as follows:

(6) $$\begin{array}{c} J\left(\omega ,b\right)=\frac{1}{m}\overset{m}{\underset{i=1}{\sum }}\left(-y\ln a+\left(1-y\right)\ln\left(1-a\right)\right). \end{array}$$

In equation (6), *a* denotes the probability that is calculated by Logistic regression. *y* represents the label of this sample, and there is a total of *m* samples. The aim for Logistic regression is to reduce the outcome of function (6) as much as possible via iteration. Through the use of multiple samples to fit the Logistic regression model for several times, the important features can be filtered out. After the above processes are completed, we obtain the matrix *A*_2_ and sort all of the matrices obtained in this step on 10 cancers into corresponding *S* subsets. Finally, we validate the existing genes and form a biomarker module through meta-analysis in a biomedical field.

### 2.4. Performance Evaluation

Four evaluation indexes are applied to evaluate method performance. A confusion matrix is used to compute and output four types of records. False Negative (FN) represents the number of positive samples predicted as negative samples; False Positive (FP) represents the number of negative samples predicted as positive samples; True Negative (TN) represents the number of negative samples predicted as negative samples; True Positive (TP) represents the number of positive samples predicted as positive samples. The following equations demonstrate the calculation of common indicators based on TP, TN, FP, and FN:

(7) $$\begin{array}{c} \text{Accuracy}:\text{ACC}=\frac{\text{TP}+\text{TN}}{\text{TP}+\text{FN}+\text{FP}+\text{FN}},\\ \text{F}1‐\text{score}:F_{1}=\frac{2\text{TP}}{2\text{TP}+\text{FP}+\text{FN}},\\ \text{Precision}:\text{PRE}=\frac{\text{TP}}{\text{TP}+\text{FP}}. \end{array}$$

Heat maps are presented to test the validation of selected genomic biomarkers of the FRL model. The efficiency level of features is reflected in the sharp edges of the heat map. The heat maps are constructed based on the rationale of the Euclidean distance with the ggplot2 package on the R platform. By clustering measurements of samples in Euclidean distance, the clustering results of samples are obtained. *X*_*MN*_ and *Y*_*MN*_ denote two matrices, in which the rows and columns are indexed by *i* and *j*. The calculation of Euclidean distance is presented as follows:

(8) $$\begin{array}{c} X_{MN}=\left[\begin{array}{c} \;\begin{array}{ccc} x_{11} & x_{12} & \cdots \\ x_{21} & x_{22} & \cdots \\ \vdots & \vdots & \cdots \end{array}\;\begin{array}{c} x_{1n}\\ x_{2n}\\ \vdots \end{array}\\ \begin{array}{ccc} x_{i1} & x_{i2} & \cdots \\ \vdots & \vdots & \cdots \\ \;x_{m1} & x_{m2} & \cdots \end{array}\;\begin{array}{c} x_{\text{in}}\\ \vdots \\ x_{mn} \end{array} \end{array}\right],\\ Y_{MN}=\left[\begin{array}{c} \;\\ \begin{array}{ccc} y_{11} & y_{12} & \cdots \\ \vdots & \vdots & \cdots \\ \;y_{m1} & y_{m2} & \cdots \end{array}\;\begin{array}{c} y_{1j}\\ y_{2j}\\ y_{mj} \end{array}\begin{array}{c} \cdots \\ \cdots \\ \cdots \end{array}\begin{array}{c} y_{1n}\\ \vdots \\ y_{mn} \end{array} \end{array}\right],\\ d\left(x_{i},y_{j}\right)=\sqrt{{\left\|x_{i}\right\|}^{2}+{\left\|y_{j}\right\|}^{2}+2x_{i}y_{j}^{T}.} \end{array}$$

## 3. Results

### 3.1. Validation for Oncology Datasets via Diverse Indicators

The first column in Figure 1 displays the initial dataset and the same dataset that has been processed by the RMA method. Compared with the initial dataset, the pretreated datasets obviously exhibit high concentrations and are prone to discover an intrinsicl relationship among genes.

**Figure 2:**
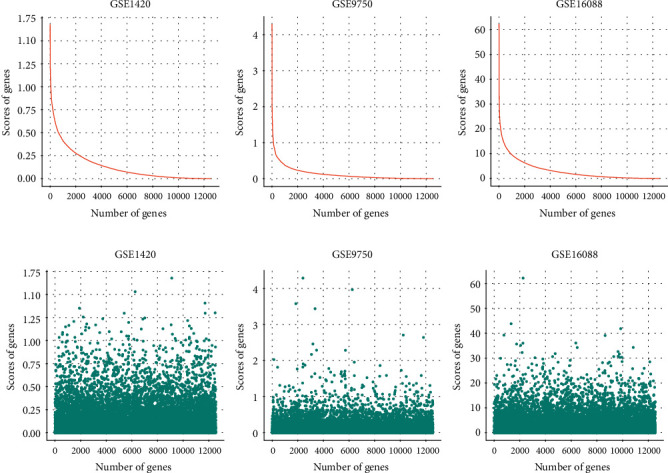
This figure directly displays the scores of genes in GSE1420, GSE9750, and GSE16088. (a) Image using a descending order to show the scores. (b) Original scores are shown in a scatter plot. The x axis represents the number of genes, while the y axis expresses the scores of genes.

Figure 2(a) exhibits the Fisher score values in a descending order, from which we can observe an obvious inflection point in each subimage.  Figure 2(b) presents the distribution of genes with diverse scores in a more visible way. It is obvious that the genes with high scores only account for a tiny fraction among the whole dataset. As listed in Table 2, only a tiny quantity of genes remain, which demonstrates that Fisher score can rapidly and effectively reduce the dimensionality.

The 2-NUM column in Table 2 displays the results for the step of feature selection in the embedded layer. This outcome has higher accuracy and precision parameters than those in the previous filter step. A series of genes are consequently selected from the recurring data, and the ultimate output is shown below in Table 3. The relevant performance of each subset is examined. Through literature review and spatial analysis of functional enrichment and functional parameters, a biomarker module is formed.

**Figure 3:**
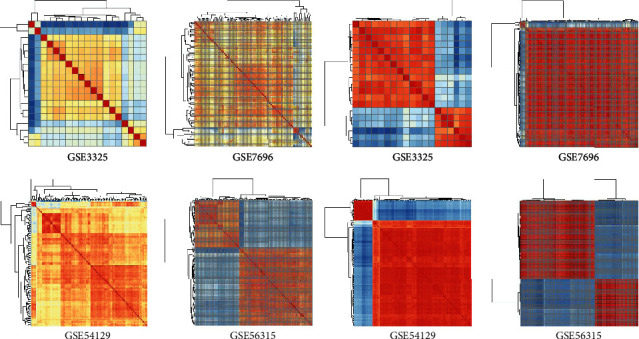
(a) Heat map of original matrix. (b) Heat map of the matrix that uses the Euclidean distance to cluster.

To validate the capability of the biomarker module, Euclidean distance matrices are adopted for clustering based on GSE3325, GSE7696, GSE54129, and GSE56315. We can directly observe that the subset obtained after feature selection clearly delimits the area (Figure 3). Thus, the final subset can be key features to represent each cancer dataset.

**Figure 4:**
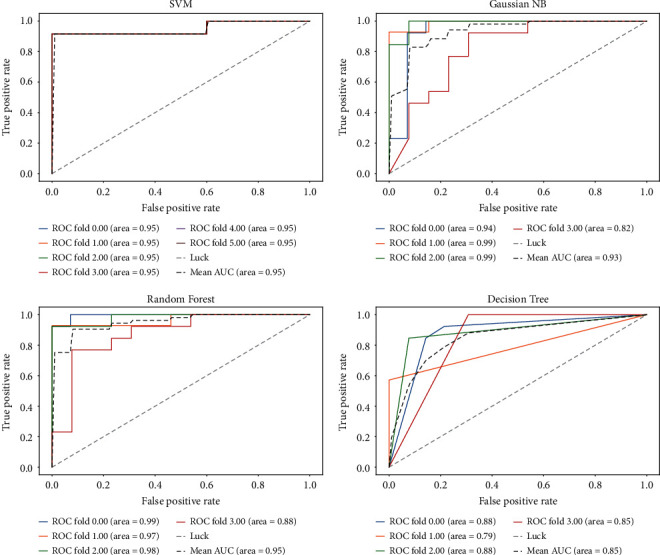
Esophageal cancer datasets (GSE1420, GSE23400) are used for experiments. GSE1420 is used as a training set, whereas GSE23400 is used as the test set. The ROC curve displays the generalization results.

To prove the validity of the FRL feature selection method on a single cancer, the diverse datasets of esophageal cancer (GSE1420, GSE23400) are chosen as representative datasets. GSE1420 is treated as the training set, and GSE23400 [37] is treated as the test set. We use genes selected by FRL on the esophageal dataset to examine the indicators of generalization ability through four main classifiers.  Figure 4 shows the receiver operating characteristic (ROC) curve and the area under the ROC curve (AUC), which is an effective and intuitive measure for evaluating feature selection classification performance. The AUC is more than 93% on three classifiers and the average AUC reaches 92%, obviously indicating that the generalization ability of the biomarker module is satisfied.

**Figure 5:**
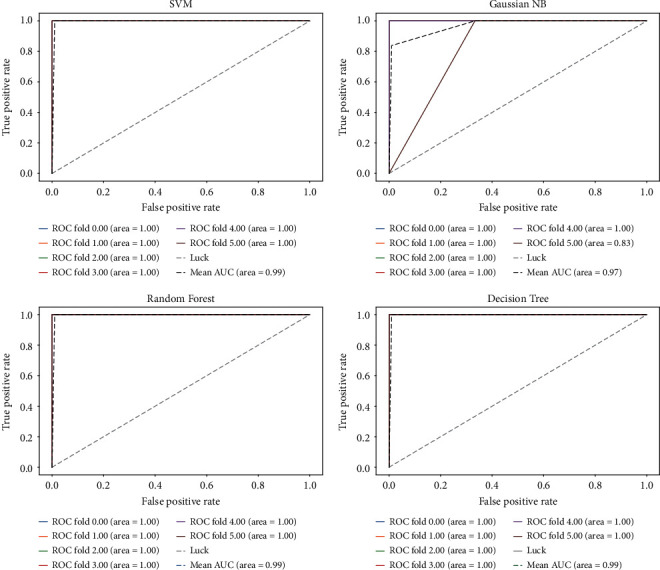
The results of another esophageal dataset (GSE26886) in the form of the ROC curve by FRL are visualized.

In addition, we build an isolated environment by dividing another dataset of esophageal cancer (GSE26886) into a test set and a training set. 35% of the samples in GSE26886 [38] are randomly chosen to construct the training set. The remaining samples are utilized as the test set.  Figure 5 presents the ROC curve of each fold. Three of the classifiers reach a value of 0.99 on areas under the ROC curve (AUC), and the average Gini index on four classifiers is 0.97, which effectively implies that the potential genomic biomarkers selected by FRL may have strong associations with cancers.

**Figure 6:**
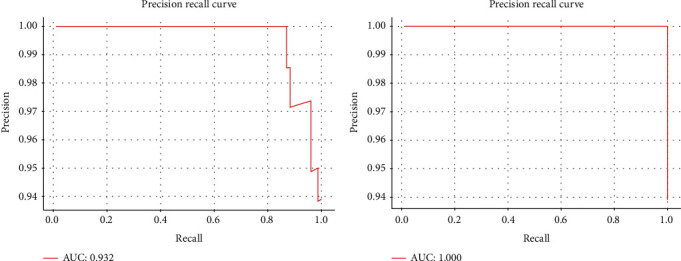
(a) The P-R curve of classification results by the five approved genetic biomarkers on GSE65194. (b) The P-R curve of classification results by potential genetic biomarkers selected by FRL on GSE65194.

Breast cancer (GSE65194) is chosen as another representative to examine the validation of FRL classification performance. *CREBBP*, *EP300*, *ESR1*, *GATA3*, and *MYC* are well-known genetic biomarkers and mutate frequently in breast tumors [39]. These five approved genetic biomarkers on breast cancer are compared with the same amount of potential biomarkers selected by FRL on GSE65194.  Figure 6 displays the Precision-Recall (P-R) curve. The AUC of the five known biomarkers is 93.2%. The AUC of FRL can be 6.8% higher than those on the five mentioned biomarkers. It is meaningful and feasible to develop further clinical experimental verification on potential genomic biomarkers selected by FRL.

### 3.2. Comparisons between FRL and Other Feature Selection Methods

**Figure 7:**
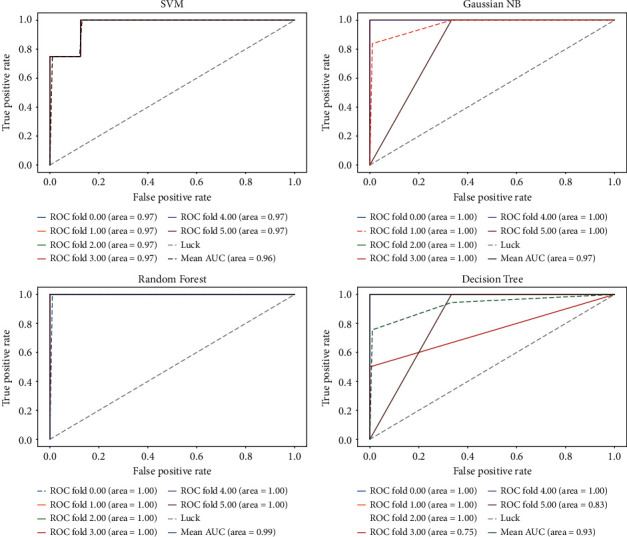
The results of another esophageal dataset (GSE26886) in the form of an ROC curve by Limma are visualized.

To compare with the differential expression analysis tool GEO2R based on Limma, esophageal cancer (GSE26886) is chosen as the representative.  Figure 7 displays the classification results on Limma by ROC curve in each fold, and the average AUC is 96.25%. It is 2.25% lower than the genes selected by FRL. On classification precision, the FRL algorithm can reach 95.56%, while Limma can only reach 91.11%. In order to obtain persuasive data, 10-fold cross validation is looped 20 times. The average ACC and F1-score of FRL are 96.24% and 96.37%, respectively. And the average ACC and F1-score of Limma are 96.04% and 95.91%, respectively. FRL is 0.2% and 0.46% superior to Limma. When looped 100 times, the metrics of FRL on ACC reaches 96.32% and can still reach 0.1759% higher than Limma. It is obvious that FRL has greater classification precision than the current differential expression analysis tool GEO2R based on the Limma algorithm on the GEO platform.

**Figure 8:**
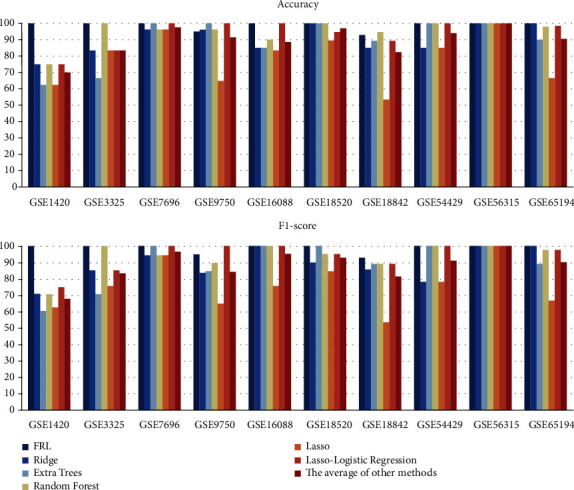
Comparisons on ACC and F1-score between FRL, Ridge regression (Ridge), Extremely randomized trees (Extra Trees), Random forest, Lasso, and Lasso-Logistic regression.

The following five methods are used for making comparisons with the FRL feature selection algorithm in terms of their ACCs and F1-scores (Figure 8): Ridge regression (Ridge), Extremely randomized trees (Extra Trees), Random Forest, Lasso, and Lasso-Logistic regression. Since there are only a few genes that have significance to carcinogenesis and can be regarded as biomarkers, by repeated experiments, we found that when the context confines the size of subset to approximately 80, it can achieve perfect classification capability. To follow variable-controlling approaches, 78 is chosen as the standard dimensionality for genes in all the subsets for all the participating methods being used as a comparison.

Table 4 lists the final prediction results of the aforementioned methods. Focusing on certain particularly low-quality datasets, such as GSE1420 (esophageal) and GSE18842 (ovarian), we summarize that the ACCs of FRL are 30% and 17.622%, respectively. These values are much higher than those of the other traditional algorithms. Regarding the average statistics (Table 4), it is clear that FRL is 10.01% and 10.428% superior to other traditional methods in terms of their average ACC and F1-score, respectively. Compared to the Lasso-Logistic regression, which is a current principal feature selection method for effective dimensionality reduction, FRL can be 5.464% and 4.534% higher than it on ACC and F1-score, respectively.

As GSE18842 (ovarian) has the highest dimensionality after the selection in a filter layer and may consume the longest time in the embedded layer, FRL and the abovementioned five methods are applied on GSE18842 (ovarian) to test the time consumed in the whole feature selection process. In order to follow variable-controlling approaches, the final subset dimensionality is restricted to 78 for all methods. As data shown in Table 5, FRL consumes only 79.619 s and can be at least three times faster than the second fastest method Lasso. However, Lasso displays low performance on ACC and F1-score. Compared to the method with approximate ACC, FRL calculates 52 times faster than Random Forest. Obviously, FRL can greatly decrease computation time, accelerate computation speed, and improve computation efficiency.

In general, an obvious and intuitive conclusion is that FRL exhibits better performance relative to current website analysis tools and other traditional feature selection methods. Compared with FRL, the conventional feature selection algorithms extract and establish unreliable and frail subsets, which may result from inappropriate performance of metrics, unbalanced labels for the dataset, and low noise immunity in the models themselves. For the remaining tested datasets, FRL also shows unique robustness, high precision, and stable classification capability.

### 3.3. A Functional Analysis of the Biomarker Module

The 19 genes in the biomarker module are as follows: *ALPI*, *AMACR*, *ANKHD1*, *ARHGAP44*, *ARHGEF15*, *ARHGEF26*, *ATXN8OS*, *CRISP3*, *HOPX*, *HSPB8*, *LSM7*, *MAFB*, *NGRN*, *PPP3R1*, *RDH5*, *SLC5A1*, *SPARC*, *SPRR3*, and *TCTN2*. Initially, we corroborate genes in the biomarker module through the following four approaches: CCLE (the Cancer Cell Line Encyclopedia, https://portals.broadinstitute.org/ccle), COSMIC (the Catalogue Of Somatic Mutations In Cancer, https://cancer.sanger.ac.uk/cosmic), NCG (the Network of Cancer Genes, https://ncg.kcl.ac.uk/index.php), and literature reviews. To depict the hereditary characteristics of cancer cells, the CCLE project has cooperated with the Broad Institute, Dana Farber Cancer Institute, and Novartis Institute. CCLE exhibits expression of genes in diverse tumor cell lines, and all the genes in our biomarker module can be found in it. COSMIC has been a neutral worldwide reference standard as it provides a comprehensive and detailed introduction for over 700 mutated genes. [40, 41]. The NCG is a database of tumor-driven genes and includes cancer information, orthology, and gene expressions in normal tissues. Furthermore, the literature is also searched to confirm the validation of the 19 genes in total, which introduces the detailed function and potential carcinogenic pathways of the above 19 genes.  Table 6 apparently shows associations between the biomarker module and various cancers.

**Figure 9:**
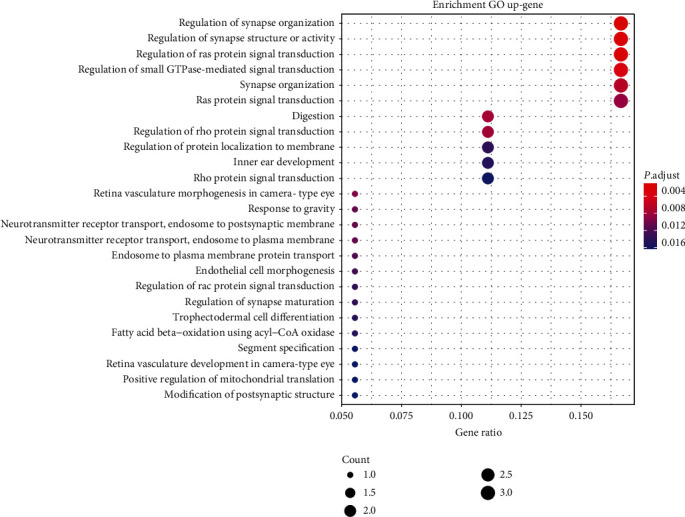
25 pathways of GO analysis on 19 selected genes. The selected genes are mainly enriched in the following functional pathways: regulation of synapse organization, regulation of synapse structure or activity, and regulation of Ras protein signal transduction (P<0.0015).

To further explore the basic biological functions of the biomarker module, cluster-Profiler from the R platform is used to perform a Gene Ontology (GO) analysis. The results concentrate in the range of *P* < 0.05, as shown in Figure 9. *P* < 0.05 is a statistics standard to denote that selected objects own significant difference. These 19 genes are mainly enriched with these three functional pathways: regulation of synapse organization, regulation of synapse structure or activity, and regulation of Ras protein signal transduction (*P* < 0.0015). These pathways are relative to the regulation of cellular component organizations and related protein expressions, which are consistent with the characteristics that cancer cells perform invasive and expansionary growth to the surrounding tissue. More detailed statistics are provided in the supplementary materials.

Through analysis of the biomarker module, we classify the genes into two main categories. The initial category, containing *ANKHD1*, *ATXN8OS*, *HSPB8*, *LSM7*, *MAFB*, and *TCTN2*, is involved in the direct regulation of cancer cell proliferation, apoptosis, and cancerization. We discovered that a single biomarker in this category can activate various signaling pathways in different cancers. For instance, *ANKHD1* is an ankyrin-repeat-containing gene involved in the regulation of a variety of cellular functions, including transcription, cell cycling, ion channels, cell survival, and cell signaling pathways. *ANKHD1* is expressed in the cytoplasm or nuclei of different tissues. In prostate cancer cell lines, *ANKHD1* is a positive regulator of *YAP1* and promotes cell growth and cell cycle progression by promoting cyclin A [42]. In the K562 line of leukemia cells, *ANKHD1* acts as a skeleton protein and affects its malignant phenotype by interacting with *SHP2* [43]. In human multiple myeloma cell lines, *ANKHD1* can act on the promoter region of the cyclin-dependent kinase inhibitor p21 to upregulate the proliferation of multiple myeloma cells and affect the cell cycle progression [44]. Another example is *HSPB8*. As a gene involved in the heat shock protein family, *HSPB8* is ubiquitously expressed in a variety of human tissues. In triple-positive hormone-sensitive breast cancer cell lines (MCF-7), *HSPB8* regulates the proliferation and reduction of the migratory ability for MCF-7 cells [45]. In ovarian cancer cell lines, the downregulation of *HSPB8* positively directs the migration progress of the transforming growth factor alpha (TGF-*α*) for ovarian cancer cells [46]. *HSPB8* is detected to be overexpressed in gastric cancer, and it regulates the proliferation and apoptosis progress of gastric cells by activating the ERK-CREB signaling [47].

The second category contains protein-coding genes, namely, *ALPI*, *ARHGEF15*, *ARHGEF26*, *SLC5A1*, *AMACR*, *ARHGAP44*, *CRISP3*, *HOPX*, *NGRN*, *RDH5*, *SPRR3*, and *PPP3R1*, which indirectly play significant roles in the regulation progress of proteins. Mutations in these genes impact protein expression levels and further lead to the development of prostate, breast, and colorectal cancers, as well as other cancer types. For example, *ARHGEF15* regulates the activation of Rho family proteins. As essential signaling molecules, Rho family proteins modulate gene expression progress, cell motility progress, cell cycles, and other processes by regulating downstream molecules such as p21-activated kinase (PAK) and the myosin-binding subunit of myosin phosphatase (MYPT1) [48]. The transcripts of *CRISP3* are widespread in human glands such as the prostate. *CRISP3* induces the abundant changes in the cell adhesion protein subsets *Lasp1* and *TJP1*, which are included both in in vitro and in vivo environments, and *CRISP3* can therefore promote the development of tumors in the prostate [49]. The overexpression of *HOPX*, upregulation of p21, and downregulation of cyclin D1 and *CDK4* regulate the progress of migration and invasion of MDA-MB-468 cells to modulate tumor growth of the breast [50]. Colorectal cancer (CRC) is an example where the expression of *SPRR3* promotes the binding between PCAT18 and miR-759 and therefore restores a portion of the proliferation and invasion capabilities of CRC cells [51]. The combination of GO analysis and the literature review directly displays the connection between the proposed biomarker module and diverse cancers.

## 4. Discussion

The high cost and low reproducibility of microarray experiments make it arduous for experimental researchers to identify common genomic biomarkers of the same type of cancer [52]. Research reflects that tumors with similar phenotypes or representing the same type of cancer can have diverse responses to the same treatment, which may result from differences in gene expression [53]. By combining all the separate cancer datasets described above, highly universal clues can be summarized and missing important clustering details can be avoided, thereby enhancing the accuracy of the algorithm as well as revealing the gene expression mechanism [54].

The first contribution of this paper is the introduction of the FRL feature selection method. The FRL framework can effectively screen out robust genes with high accuracy. The traditional filter method, which aims at acquiring and removing redundant features, is not useful for extracting robust subsets of cancers. This paper uses Fisher score to screen subsets in the filter layer, and it mainly depends on human judgment to choose the optimal subset. A deficiency in the filter layer is the threshold setting requirement. In practice, the subset selected by human judgment will differ from the optimal subset. Therefore, in the future, we could explore more algorithms to obtain accurate calculations of the threshold, thereby enhancing flexibility and adaptability. In addition, Fisher score is effective for selecting high-score features. Thus, it is possible that some biomarkers with low scores are ignored in the selection process. In the future, we can pay more attention to low-score genes, which would be beneficial for the thorough exploration of certain cancers.

The second contribution of this paper is the effective extraction of genes to form a biomarker module based on 10 cancers associated with high mortality. Cluster studies can be beneficial in revealing the core mechanisms of high-mortality cancers [53]. Through GO analysis and multiple literature review, all the genes in this module have been verified to be related to diverse cancers. Furthermore, details of some of the pathways involving these genes have been obtained. The biomarker module tends to provide a range of genes that have great performances. It could overcome the difficulty of single genes, i.e., it is hard for a single gene to recur on gene chips; therefore, this narrows down the selection scope for clinical researchers. However, there is still a limitation for directly applying potential biomarkers in our module on a single type of cancer. With comprehensive information obtained from cluster studies on multiple datasets, we can combine calculated data with clinical data, whole-genome sequencing, and the use of gene expression atlases to further explore the types and sites of gene mutations for single types of cancer in the future, which can attach great significance to the development of the health evaluation of ultra-early cancers associated with high mortality, the evaluation of radiotherapy efficacy, the prediction of the efficacy of target drugs, and monitoring for early postoperative recurrence of single types of cancers in precision medicine. Furthermore, we can upload our biomarker module data to a public database for other researchers to download, which can overcome drawbacks such as data deficiency and accelerate the identification process of all cancer biomarkers in *Homo sapiens*.

## 5. Conclusions

We present an integrative feature selection algorithm called FRL, which employs Fisher score, Recursive feature elimination, and Logistic regression (FRL). It has greater precision performance than differential expression analysis tools based on Limma and five traditional feature selection methods. Time consumption has also been reduced through comparison with the abovementioned five methods. With the help of this method, we screened 19 genes from a total of 189224 genes in 10 high-mortality-cancer datasets to form a biomarker module. Via GO analysis and multiple meta-analysis in the biological field, all genes in this module are proven capable of serving as potential biomarkers of the regulation of cancer cellular component organization or related protein expressions, which corresponds to the characteristics that cancer cells perform invasive and expansionary growth to the surrounding tissue. This module is beneficial to health assessment, drug screening, and targeted therapy. In addition, the selected potential biomarker module can supply information on the development of cancers with high mortality, which assists in precision medicine.

## Figures and Tables

**Procedure 1:**
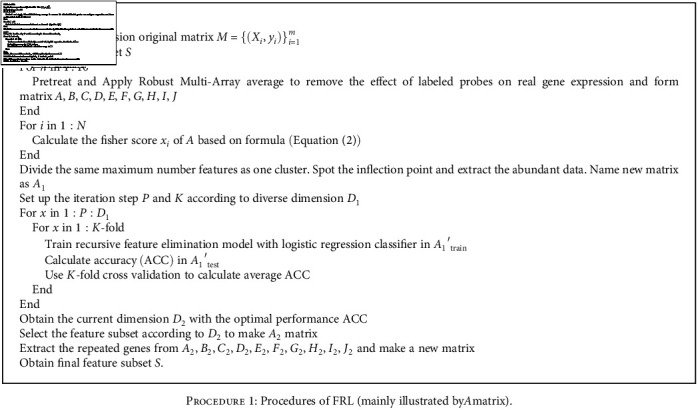
Procedures of FRL (mainly illustrated by*A*matrix).

**Table 1:** Introduction of the gene expression data on 10 cancers.

ID	Dataset	Cancer	Normal	References
GSE1420	Esophageal	8	16	[22]
GSE3325	Prostate	6	13	[23]
GSE7696	Glioblastoma	80	4	[24, 25]
GSE9750	Cervical	33	24	[26]
GSE16088	Osteosarcoma	14	6	[27]
GSE18520	Ovarian	53	10	[28]
GSE18842	Lung	46	45	[29]
GSE54129	Gastric	111	21	[30]
GSE56315	Diffuse large B-cell lymphoma	55	33	[31, 32]
GSE65194	Breast	153	11	[33, 34]

**Table 2:** Performance measures of the original dataset, the first round, and the second round.

ID	0-ACC	0-NUM	1-ACC	1-F1-score	1-NUM	2-ACC	2-F1-score	2-NUM
GSE1420	62.50%	12549	100%	100%	1803	100%	100%	78
GSE3325	83.33%	21654	83.33%	75.76%	1302	100%	100%	69
GSE7696	96.15%	21654	100%	100%	1803	100%	100%	65
GSE9750	65.00%	21654	90.00%	89.52%	1403	100%	100%	78
GSE16088	66.67%	12549	83.33%	85.19%	2003	100%	100%	78
GSE18520	89.47%	21653	100%	100%	753	100%	100%	50
GSE18842	57.14%	21654	71.43%	69.60%	3453	96.43%	96.42%	77
GSE54129	85.00%	21654	85.00%	78.11%	1011	100%	100%	77
GSE56315	96.30%	21654	100%	100%	1603	100%	100%	78
GSE65194	94.00%	21654	100%	100%	801	100%	100%	79

**Table 3:** Performance indicators on the final subsets for diverse cancers.

ID	Dataset	Gene-num	PRE	ACC	F1-score
GSE1420	Esophageal	5	89.58%	87.50%	86.82%
GSE3325	Prostate	2	100%	100%	100%
GSE7696	Glioblastoma	3	92.46%	96.15%	94.27%
GSE9750	Cervical	5	92.22%	90%	90.21%
GSE16088	Osteosarcoma	4	100%	100%	100%
GSE18520	Ovarian	2	96.49%	94.74%	95.18%
GSE18842	Lung	3	91.29%	89.29%	89.24%
GSE54129	Gastric	3	100%	100%	100%
GSE56315	Diffuse large B-cell lymphoma	4	100%	100%	100%
GSE65194	Breast	3	100%	100%	100%

**Table 4:** Comparisons between FRL and other five feature selection algorithms.

Indicators	FRL		Ridge		Extra Trees		Random Forest		Lasso		Lasso-Logistic regression		Average of the other methods	
	ACC	F1-score	ACC	F1-score	ACC	F1-score	ACC	F1-score	ACC	F1-score	ACC	F1-score	ACC	F1-score
GSE1420	100	100	75	70.83	62.5	60.45	75	70.83	62.5	62.5	75	75	70	67.92
GSE3325	100	100	83.33	85.19	66.67	70.83	100	100	83.33	75.76	83.33	85.19	83.33	83.39
GSE7696	100	100	96.15	94.27	100	100	96.15	94.27	96.15	94.27	100	100	97.69	96.56
GSE9750	100	94.9	96.15	83.73	100	84.7	96.15	89.52	65	65	100	100	91.46	84.59
GSE16088	95	100	85	100	85	100	90	100	83.33	75.76	100	100	88.67	95.15
GSE18520	100	100	100	90	100	100	100	95.18	89.47	84.5	94.74	95.18	96.84	92.97
GSE18842	100	92.86	85	85.57	95	78.57	94.74	89.24	53.57	53.57	89.29	89.24	82.38	81.37
GSE54129	100	100	85	78.11	100	100	100	100	85	78.11	100	100	94	91.24
GSE56315	100	100	100	100	100	100	100	100	100	100	100	100	100	100
GSE65194	100	100	100	100	90	89.05	98	97.81	66.67	66.67	98	97.81	90.53	90.27
Average	99.5	98.776	90.563	88.77	89.917	88.36	95.004	93.685	78.502	75.614	94.036	94.242	89.49	88.348

**Table 5:** Comparisons between FRL and five other feature selection algorithms on time consumption.

Algorithm	Time consumption
FRL	79.619 s
Ridge	3328.9418 s
Extra Trees	2690.1589s
Random Forest	4148.396 s
Lasso	263.944 s
Lasso-Logistic regression	264.042 s

**Table 6:** Metavalidation results of selected genes.

	CCLE	Literature	COSMIC	NCG
ALPI	**√**	**√**	**√**	
AMACR	**√**	**√**	**√**	
ANKHD1	**√**	**√**	**√**	
ARHGAP44	**√**	**√**	**√**	
ARHGEF15	**√**	**√**	**√**	
ARHGEF26	**√**	**√**	**√**	
ATXN8OS	**√**	**√**		
CRISP3	**√**	**√**	**√**	
HOPX	**√**	**√**	**√**	
HSPB8	**√**	**√**	**√**	**√**
LSM7	**√**	**√**	**√**	
MAFB	**√**	**√**	**√**	**√**
NGRN	**√**	**√**	**√**	
PPP3R1	**√**	**√**	**√**	
RDH5	**√**	**√**	**√**	
SLC5A1	**√**	**√**	**√**	
SPARC	**√**	**√**	**√**	
SPRR3	**√**	**√**	**√**	
TCTN2	**√**	**√**	**√**	

## Data Availability

The datasets for this study (GSE1420, GSE3325, GSE7696, GSE9750, GSE16088, GSE18520, GSE18842, GSE54129, GSE56315, GSE65194, GSE26886 and GSE23400) can be found in https://www.ncbi.nlm.nih.gov/. The code of FRL can be found in https://github.com/jianan-kristine/code.git.

## Supplementary Materials

Supplementary 1 Additional 1 presents the values of performance indicators of the selected model for esophageal cancer (GSE26886).

Supplementary 2 Additional 2 shows more detailed statistics for the *P* value which is less than 0.01 of GO analysis on the functional path in Figure 9.
