# Supplementary material for: FRL: An Integrative Feature Selection Algorithm Based on the Fisher Score, Recursive Feature Elimination, and Logistic Regression to Identify Potential Genomic Biomarkers
Source: Biomed Res Int. 2021 Jun 12;2021:4312850. doi: 10.1155/2021/4312850 (PMC8218915; doi:10.1155/2021/4312850)
Supplement: Supplementary 1 — Additional 1 presents the values of performance indicators of the selected model for esophageal cancer (GSE26886). [file 4312850.f1.docx]

| Additional 1: Performance indicators of selected model on Esophageal (GSE26886) | | | | |
| --- | --- | --- | --- | --- |
| Method | ACC | F1-score | PRE | REC |
| SVM | 100% | 100% | 100% | 100% |
| Gaussian NB | 90% | 89.67% | 91.43% | 90% |
| Random Forest | 100% | 100% | 100% | 100% |
| Decision Tree | 100% | 100% | 100% | 100% |
