# Supplementary material for: FRL: An Integrative Feature Selection Algorithm Based on the Fisher Score, Recursive Feature Elimination, and Logistic Regression to Identify Potential Genomic Biomarkers
Source: Biomed Res Int. 2021 Jun 12;2021:4312850. doi: 10.1155/2021/4312850 (PMC8218915; doi:10.1155/2021/4312850)
Supplement: Supplementary 2 — Additional 2 shows more detailed statistics for the P value which is less than 0.01 of GO analysis on the functional path in Figure 9. [file 4312850.f2.docx]

| Additional 2: Part of the GO analysis on functional path (P<0.01) | | |
| --- | --- | --- |
| ID | Description | P-value |
| GO:0050807 | regulation of synapse organization | 0.001125629 |
| GO:0050803 | regulation of synapse structure or activity | 0.001264693 |
| GO:0046578 | regulation of Ras protein signal transduction | 0.001448854 |
| GO:0051056 | regulation of small GTPase mediated signal transduction | 0.003921781 |
| GO:0050808 | synapse organization | 0.006622872 |
| GO:0007586 | digestion | 0.007787318 |
| GO:0035023 | regulation of Rho protein signal transduction | 0.007895684 |
| GO:0007265 | Ras protein signal transduction | 0.008564694 |
| GO:0061299 | retina vasculature morphogenesis in camera-type eye | 0.009601719 |
